# Genomic Screening Identifies Individuals at High Risk for Hereditary Transthyretin Amyloidosis

**DOI:** 10.3390/jpm11010049

**Published:** 2021-01-15

**Authors:** Emily R. Soper, Sabrina A. Suckiel, Giovanna T. Braganza, Amy R. Kontorovich, Eimear E. Kenny, Noura S. Abul-Husn

**Affiliations:** 1 The Institute for Genomic Health, Icahn School of Medicine at Mount Sinai, New York, NY 10029, USA; emily.soper@mssm.edu (E.R.S.); sabrina.suckiel@mssm.edu (S.A.S.); giovanna.braganza@mssm.edu (G.T.B.); eimear.kenny@mssm.edu (E.E.K.); 2 Department of Medicine, Icahn School of Medicine at Mount Sinai, New York, NY 10029, USA; 3 The Zena and Michael A. Wiener Cardiovascular Institute, Icahn School of Medicine, New York, NY 10029, USA; amy.kontorovich@mssm.edu; 4 Department of Genetics and Genomic Sciences, Icahn School of Medicine at Mount Sinai, New York, NY 10029, USA

**Keywords:** cardiac amyloidosis, genomic screening, heart failure, hereditary transthyretin amyloidosis, TTR

## Abstract

The TTR V142I variant associated with hereditary transthyretin amyloidosis (hATTR) is present in up to 4% of African American (AA) and 1% of Hispanic/Latinx (HL) individuals and increases risk for heart failure. Delayed and missed diagnoses could potentiate health disparities in these populations. We evaluated whether population-based genomic screening could effectively identify individuals at risk for hATTR and prompt initiation of risk management. We identified participants of the Bio*Me* Biobank in New York City who received TTR V142I results through a pilot genomic screening program. We performed a retrospective medical record review to evaluate for the presence hATTR-related systemic features, uptake of recommended follow-up, and short-term outcomes. Thirty-two AA (*N* = 17) and HL (*N* = 15) individuals received a TTR V142I result (median age 57, 81% female). None had a previous diagnosis of hATTR. Eighteen (56%) had hATTR-related systemic features, including 4 (13%) with heart failure, 10 (31%) with carpal tunnel syndrome, and 10 (31%) with spinal stenosis. Eighteen (56%) pursued follow-up with a cardiologist within 8 months. One person received a diagnosis of hATTR. Thus, we found that the majority of V142I-positive individuals had hATTR-related systemic features at the time of result disclosure, including well-described red flags. Genomic screening can help identify hATTR risk and guide management early on, avoiding potential delays in diagnosis and treatment.

## 1. Introduction

Population-based genomic screening is an emerging approach to uncover disease risk in unsuspecting individuals [1]. There is increasing evidence that genomic screening may identify and improve outcomes for certain medically actionable conditions for which preventive measures and/or effective treatments are available [2,3]. Genomic screening programs, typically performed in the setting of large health system clinics [4], biobanks [2], or public health initiatives [3], may choose to return medically actionable findings with varying degrees of clinical utility. Recent studies have found that most individuals at genomic risk for medically actionable conditions are unaware of their risk [5,6,7], and that the majority of individuals informed of such risk will undergo recommended surveillance or risk-reducing interventions [2,8]. Therefore, genomic screening holds the promise of identifying preventable and treatable diseases that may be otherwise missed in routine clinical care.

Hereditary transthyretin amyloidosis (hATTR) is an under-recognized systemic disease caused by pathogenic variants in the transthyretin (TTR) gene [9,10]. Such variants cause misfolding of the tetrameric transthyretin protein complex, resulting in the extracellular deposition and accumulation of insoluble amyloid fibrils. hATTR is transmitted in an autosomal dominant manner and with incomplete penetrance. Due to the nonspecific and progressive nature of its symptoms, hATTR diagnosis is frequently delayed or missed [11,12,13]. V142I is the most common pathogenic TTR variant worldwide, with recent prevalence estimates of 1 in 330 individuals [14,15]. Up to 4% of African Americans (AA) in the U.S. harbor the V142I variant [16], and V142I was also recently shown to be prevalent (1%) in Hispanic/Latinx (HL) populations in New York City [17].

V142I is classically associated with cardiac amyloidosis, resulting in an increased risk for cardiomyopathy and heart failure (HF) [18,19]. However, there is wide variability in the clinical spectrum, with many patients experiencing overlapping cardiac, neurologic, and other phenotypes [20,21]. Certain systemic “red flags”, including carpal tunnel syndrome (CTS) and spinal stenosis (SS), raise clinical suspicion for hATTR and have been found to precede TTR-associated cardiac amyloidosis by 5–10 years [22,23,24,25]. Identifying patients with hATTR early in the disease course is key to realizing the benefits of newly approved targeted therapies to suppress and stabilize transthyretin [26,27,28]. Tafamidis, an FDA approved medication for hATTR patients with cardiac amyloidosis [29], prevents the accumulation of amyloid fibrils, but it does not reduce existing amyloid deposits. Given the high frequency of V142I among AAs and HLs, overlooked or delayed diagnoses may miss the optimal therapeutic window and potentiate health disparities impacting these patient populations.

We recently launched a pilot program to screen for medically actionable genomic conditions in a large, ancestrally diverse biobank in New York City [30]. As part of this program, we screened eligible participants for pathogenic variants in TTR associated with hATTR. The aim of the present study was to assess whether genomic screening could effectively identify individuals at risk for hATTR and prompt their follow-up for risk management. We identified biobank participants who received TTR V142I results through the pilot genomic screening program, and we evaluated their medical records for hATTR-related systemic features, uptake of recommended follow-up, and short-term outcomes. This study highlights the potential for translating genomic risk information into the care of diverse patient populations.

## 2. Materials and Methods

### 2.1. Study Setting and Population

The Bio*Me* Biobank is an ongoing electronic health record (EHR)-linked biorepository in New York City. Over 55,000 Bio*Me* participants have been enrolled since 2007, predominantly through unselected medicine and specialty ambulatory care practices across the Mount Sinai Health System (MSHS) [5,31]. A subset of Bio*Me* participants have undergone exome sequencing and genotyping through a collaboration with the Regeneron Genetics Center [5]. In October 2018, the Bio*Me* protocol and consent were modified to include the option for participants to receive clinically confirmed, medically actionable genomic results. The present study population consisted of consenting Bio*Me* participants with available exome sequence and/or genotype data who opted to receive genomic results, and who subsequently received a TTR V142I result through a pilot genomic screening program from November 2019 through November 2020.

All participants provided written informed consent prior to participating in the Bio*Me* Biobank. This study was conducted in accordance with the Declaration of Helsinki, and the research was approved by the Icahn School of Medicine at Mount Sinai’s Institutional Review Board (HS07-0529; HS19-00942).

### 2.2. Pilot Genomic Screening Program

A pilot genomic screening program was launched in February 2019 to disclose clinically confirmed, medically actionable genomic results to Bio*Me* participants [30]. The program initially included the Centers for Disease Control and Prevention Tier 1 genomic applications (hereditary breast and ovarian cancer syndrome, Lynch syndrome, and familial hypercholesterolemia) [32]. The program was expanded in November 2019 to include hATTR, due to the high prevalence of TTR V142I in the MSHS patient population [17], the availability of non-invasive procedures for the diagnosis of cardiac amyloidosis [33], recent FDA approval of new therapies for cardiac and neuropathic transthyretin amyloidosis [26,27,28,29], and the availability of expert multidisciplinary clinical care for amyloidosis in the health system.

### 2.3. *TTR* V142I Results Disclosure

**Figure 1:**
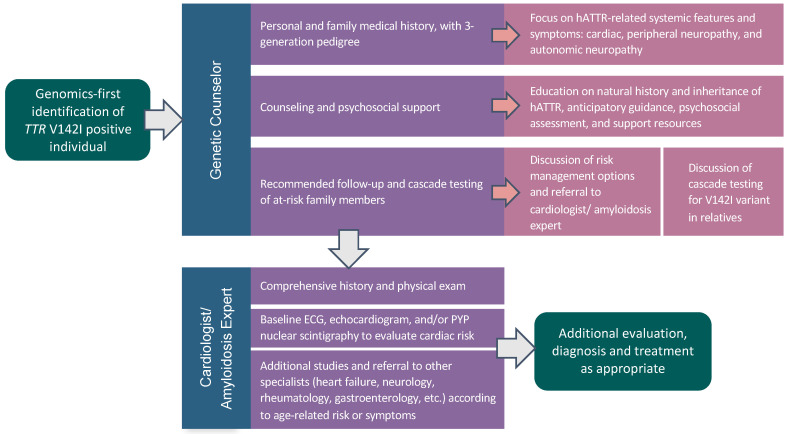
A genomics-first model for identification and guided follow-up of TTR V142I-positive individuals. After a genomics-first identification of TTR V142I, individuals underwent genetic counseling and were referred to a cardiologist/amyloidosis expert for a comprehensive assessment, initiation of surveillance, and further workup, as appropriate.

Exome sequence and genotype data from consenting Bio*Me* participants were screened for the pathogenic TTR V142I variant (c.424G>A, 18:31598655:G:A, GRCh38, NM_000371.4). Participants with a research result for TTR V142I who had opted to receive medically actionable genomic results provided a new blood sample for Sanger confirmation at Sema4, a New York State-approved and CLIA-certified clinical genetic testing laboratory [34]. Participants received clinically confirmed TTR V142I results during an in-person or telehealth result disclosure and genetic counseling visit. A genetic counselor obtained a targeted personal and family medical history, which included constructing a three-generation pedigree focused on signs and symptoms of hATTR (Figure 1). Education regarding the natural history and inheritance of hATTR was provided, with an emphasis on the non-deterministic nature of genomic risk, and the potential value of recommended follow up. A copy of the genomic result was uploaded into the participant’s EHR, and the problem list was updated to include TTR variant positive status. Participants were referred for clinical evaluation by a cardiologist (A.R.K.) with expertise in cardiovascular genetics and amyloidosis. Additional referrals to other specialties were subsequently made as needed.

### 2.4. Characteristics of V142I-Positive Individuals at Result Disclosure

Self-reported race/ethnicity groups were derived from a survey administered to participants upon enrollment into Bio*Me*, as previously described [5,31]. A list of hATTR- related phenotypes was compiled to query participants’ medical records. In addition to cardiac phenotypes, we considered peripheral neuropathy and autonomic neuropathy phenotypes that are more often described in association with other TTR pathogenic variants [21,35,36]. The list of hATTR-related phenotypes was refined through multiple iterations involving four study authors (S.A.S., E.R.S., N.S.A.-H., and A.R.K.). The final list consisted of eight systemic features: cardiac—HF, cardiomyopathy, and atrial fibrillation; peripheral neuropathy—carpal tunnel syndrome and spinal stenosis; and autonomic neuropathy—autonomic dysfunction (including gastroparesis and orthostatic hypotension), incontinence, and sexual dysfunction/impotence. In addition, we queried a list of seventeen symptoms related to these systemic features (see Supplemental Table S1). Between 1 June 2020 and 31 December 2020, a medical record review for phenotypes ascertained up to and at the time of result disclosure was carried out by a manual search of each participant’s MSHS EHR using every phenotype as a search term. Phenotypes were considered present if they appeared in any part of a provider note, imaging report, or problem list, and were recorded in a spreadsheet. An independent manual review was completed by two investigators (E.R.S. and S.A.S.), and any discrepancies were discussed and resolved by a third investigator (N.S.A.-H.). Pedigrees obtained during result disclosure were reviewed for hATTR-related features in first- or second-degree relatives (Supplemental Table S1).

### 2.5. Post-Result Disclosure Follow-Up and Outcomes

Uptake of medical recommendations and hATTR-related clinical outcomes within 5 to 14 months post-result disclosure were identified through retrospective medical record review. The EHR was assessed for relevant follow-up evaluations with any specialist in relation to TTR results. hATTR-related surveillance and procedures, including electrocardiogram (ECG), echocardiogram, technetium-99m pyrophosphate (Tc-99m-PYP) scintigraphy with single photon emission computed tomography (SPECT) imaging, nerve conduction studies, and histologic analysis of tissue for amyloid were recorded. All hATTR-related post-disclosure clinical encounters were reviewed for newly reported hATTR-related phenotypes, treatment, or diagnosis of hATTR.

### 2.6. Statistical Analysis

Results are described using frequencies, medians, and ranges. The total number of hATTR-related systemic features was compared across age (<60 years vs. ≥60 years), sex, and self-reported race/ethnicity groups (AA vs. HL) using a two-sided Fisher’s exact test. Statistical analyses were performed using GraphPad Prism v.8.2.4.

## 3. Results

In total, 33 Bio*Me* participants received a TTR V142I result through a pilot genomic screening program. One participant with a pre-existing neurological condition was excluded from the present study due to the inability to discern neuropathic features potentially associated with hATTR. Among the 32 remaining V142I-positive participants, self-reported race/ethnicity was 17 (53%) AA and 15 (47%) HL (Table 1). Median age was 57 years (range 30–79), and 26 (81%) participants were female. None had a previous diagnosis of hATTR. 

**Figure 2:**
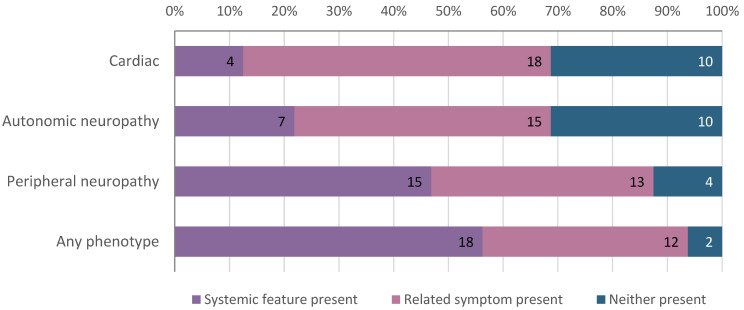
TTR V142I variant positive individuals with hereditary transthyretin amyloidosis (hATTR)-related systemic features and/or related symptoms at result disclosure. The majority of V142I variant positive individuals (56%) had at least one hATTR-related systemic feature at the time of result disclosure. Cardiac features (present in 13%) include congestive heart failure and cardiomyopathy; related symptoms include shortness of breath, dizziness, syncope, palpitations, and edema. Autonomic neuropathy features (present in 22%) include cardiac autonomic dysfunction, incontinence, and sexual dysfunction/impotence; related symptoms include nausea/vomiting, diarrhea, constipation, and loss of appetite/early satiety. Peripheral neuropathy features (present in 47%) include carpal tunnel syndrome and spinal stenosis; related symptoms include carpal tunnel related symptoms, gait/balance problems, muscle weakness, numbness/tingling, pain in extremities, back pain, and joint pain.

We queried participants’ medical records for the presence of cardiac, peripheral neuropathy, and autonomic neuropathy features of hATTR (Figure 2 and Supplemental Table S1). At the time of result disclosure, 18 (56%) participants had at least one hATTR-related systemic feature. Peripheral neuropathy was most common, present in 15 (47%) individuals (Figure 2). This was largely driven by the high prevalence of CTS (*N* = 10, 31%) and SS (*N* = 10, 31%). Five participants had lumbar stenosis, 6 had cervical stenosis, and 1 had both. Autonomic neuropathy features were noted in 7 (22%) individuals and included cardiac autonomic dysfunction (*N* = 1, 3%), incontinence (*N* = 4, 13%), and sexual dysfunction/impotence (*N* = 3, 9%). Four (13%) individuals had HF. An additional 12 (38%) participants had at least one of seventeen related symptoms in the absence of a systemic feature of hATTR (Figure 2 and Supplemental Table S1).

Fifteen (47%) V142I-positive individuals had a family history of any hATTR-related systemic feature in first- or second-degree relatives (Table 1 and Supplemental Table S1). A family history of CTS was most common, reported in 13 first- or second-degree relatives of 8 (25%) participants. Six (19%) participants reported a family history of HF in a total of 8 relatives. No participants had a known family history of hATTR.

We evaluated the uptake of recommended follow-up among participants who received TTR V142I results. Eighteen (56%) participants followed up with a cardiologist/cardiovascular geneticist (Table 2) after result disclosure. These visits occurred within 8 months of results disclosure (median 4.9 weeks). Recommended cardiac evaluation included baseline ECG and echocardiogram for all V142I-positive individuals (unless a test had been obtained in the past 12 months) and Tc-99m-PYP scintigraphy with SPECT imaging based on age and risk factors. Sixteen of the 18 individuals (89%) who followed up with a specialist underwent recommended cardiac evaluations, including ECG (*N* = 10), echocardiogram (*N* = 12), and/or Tc-99m-PYP scintigraphy (*N* = 10). An AA man with HF had findings on echocardiogram and Tc-99m-PYP scintigraphy consistent with cardiac amyloidosis, and was subsequently diagnosed with hATTR. This is the only participant with a diagnosis of hATTR at the time of this study. 

Three individuals had opportunistic histological evaluation of tissue samples for amyloid. Two HL women had histological analysis with Congo red staining of previous tissue specimens, which were negative for amyloid. The third, an AA woman with coronary artery disease, had an endomyocardial biopsy performed during a previously scheduled cardiac catheterization, which was negative for amyloid. None of these individuals had a diagnosis of hATTR at the time of this study.

Additional hATTR-related features were documented for three individuals who completed follow-up evaluations, including one individual with HF and atrial fibrillation, a second with sexual dysfunction, and a third with incontinence. With these, a total of 19 (59%) V142I-positive individuals were found to have an hATTR-related systemic feature (Supplemental Table S1). All 10 individuals with CTS had evidence of bilateral involvement. Specialists also identified additional symptoms associated with hATTR in 12 participants. In total, following a specialist’s evaluation, 31 individuals (96%) had either a systemic feature of hATTR or a related symptom.

The presence of hATTR-related systemic features was compared across age, sex, and self-reported race/ethnicity groups (Supplemental Table S2). The proportion of individuals with hATTR-related features was not significantly different in those ≥60 years vs. <60 years (*P* = 0.29), in males vs. females (*P* = 0.67), or in AA vs. HL participants (*P* = 0.49).

## 4. Discussion

hATTR is an under-recognized yet treatable disease. Accurate diagnosis early in the disease course is critical, as novel therapeutics prevent new cardiac amyloid deposition but do not ameliorate existing amyloid [28]. hATTR diagnosis can be improved by better understanding the natural history and phenotypic spectrum of disease. In November 2019, we initiated the return of pathogenic TTR variants to biobank participants through a pilot genomic screening program [30], which offers an unprecedented opportunity to examine the presence of hATTR-related systemic features in an unselected population at high genomic risk. Here, we explored the characteristics of 32 biobank participants receiving a TTR V142I result who were previously unaware of their risk. All participants were either AA or HL by self-report, populations that have been underrepresented in genomic research in the U.S. and elsewhere [37]. We found that the majority had at least one hATTR-related systemic feature at the time of their result disclosure (56%), and even more so after some underwent follow-up specialist evaluation (59%).

Penetrance of HF in V142I-positive individuals is not well defined [19,38], but the risk has been shown to increase with age [39,40]. Five individuals in our study had a diagnosis of HF, four of which were present at the time of result disclosure and one of which became clinically apparent directly following result disclosure. This is the only participant diagnosed with hATTR to date. Only one additional patient had sufficient features to prompt an opportunistic endomyocardial biopsy, which was ultimately negative. Although the penetrance of cardiac amyloid deposition in V142I has been estimated to be as high as 80% by age 70 [40], when considered from a genomics-first approach, penetrance may be much lower [19]. This suggests that routine inclusion of invasive procedures such as endomyocardial biopsies in the workup of younger and/or asymptomatic V142I-positive individuals may not be justified.

Studies of V142I have primarily centered on cardiac features, with limited focus on neuropathy [21,39,41]. Bilateral CTS and SS emerged as common phenotypes in V142I-positive individuals in our study. Though often highlighted as a feature of wild-type ATTR or with other pathogenic TTR variants [23], CTS has also been described in association with V142I [42]. A recent study found CTS in 16 of 55 V142I-positive individuals, most of whom had a known diagnosis of hATTR [21]. While SS—particularly lumbar SS—is well-recognized in wild-type ATTR [22], its association with hATTR has only been appreciated more recently [23]. Symptoms that could be attributable to an hATTR-related systemic feature were exceedingly common in V142I-positive individuals. Shortness of breath, numbness/tingling, back pain, joint pain, and gastrointestinal symptoms were all observed in over half of the participants. Many of these symptoms are non-specific and, thus, may not raise suspicion for hATTR, particularly if other associated comorbidities are present (e.g., paresthesia with diabetes) [12]. As such, these symptoms may be most appropriately used to guide workup for amyloidosis in known V142I variant positive individuals, rather than to help identify candidates for TTR genetic testing.

The current approach to TTR V142I identification typically relies on the presence of cardiac amyloidosis in a symptomatic individual. This can be detected via tissue biopsy, or more recently, non-invasive nuclear imaging, such as Tc-99m-PYP scintigraphy with corroborative monoclonal protein testing [11]. TTR genetic testing is then performed to differentiate hereditary from wild-type ATTR. Unaffected, at-risk individuals may be discovered through cascade testing of close relatives, and consensus guidelines for counseling have recently been proposed in this setting [43,44]. In April 2019, a direct-to-consumer genetic testing company 23andMe added V142I and two other common TTR variants, V50M and T80A, to its Health Risk Reports [45], allowing individuals to discover their genomic risk without the involvement of a healthcare provider. We therefore expect increasing numbers of asymptomatic individuals harboring pathogenic TTR variants to come to medical attention. There are currently no evidence-based guidelines for the management of TTR variant positive individuals. However, recommendations based on expert opinion underscore the call for early identification and multidisciplinary care of at-risk individuals so that surveillance and, if indicated, therapy would have the greatest potential for benefit [44,46]. Strategies for managing both asymptomatic and symptomatic individuals will likely evolve over time. This study helps inform a process to initiate risk management in V142I-positive individuals identified by a genomics-first approach (Figure 1).

V142I-positive individuals made medical management decisions based on their results. Over half sought follow-up care with a cardiologist within 8 months of result disclosure, and most of these individuals completed recommended cardiac evaluation. This is consistent with uptake observed in a genomic screening program for other actionable conditions, where 56% of participants with no prior knowledge of genomic risk followed up with a specialist referral and/or procedure [2]. In our study, follow-up specialist evaluations led to the identification of additional cardiac and non-cardiac phenotypes related to hATTR. This underscores the value of multidisciplinary specialty care in the evaluation of individuals at high risk of hATTR.

There are limitations to this study. We retrospectively reviewed participants’ medical records, which may not fully capture all relevant medical and family history. However, targeted information was obtained during result disclosure visits, and during subsequent specialist visits, reducing the chance of missing relevant information. This study included a small sample of mostly female biobank participants, which limits the generalizability of our findings. The timeframe of the study may have been too short for participants to complete recommended medical management, and it coincided with the peak of the COVID-19 pandemic in New York City, which may have impacted uptake of recommendations. Concomitant comorbidities, such as diabetes and hypertension, may have contributed to some of the phenotypes captured in this study. That said, it is important to recognize that such comorbidities may obscure recognition of hATTR-related phenotypes in day-to-day clinical practice, leading to missed diagnosis and/or patients experiencing a diagnostic odyssey [47].

This study helps to inform genomics-first approaches for an under-recognized condition that largely impacts AA and HL populations in the U.S. The presence of population-specific disease variants in underrepresented populations may cause health disparities [48]. Approximately 4% of AA and 1% of HL in NYC harbor the TTR V142I variant and are, thus, at increased risk for hATTR. Despite having potentially related features of hATTR, none of our study participants had prior knowledge of their genomic risk. Genomic result disclosure prompted follow-up evaluation with a specialist in over half of participants, with initiation of surveillance in most. Findings in one individual were diagnostic for hATTR, facilitating appropriate therapy. Longitudinal follow up will be necessary to improve the early identification of hATTR cardiac amyloidosis in V142I-positive individuals and to determine optimal surveillance strategies and treatment once amyloidosis is present. Further questions remain regarding the appropriate implementation of genomic screening, cost-benefit of this approach, and equitable access to recommended follow-up care.

## Figures and Tables

**Table 1:** Characteristics of TTR V142I variant positive individuals at the time of result disclosure.

Characteristics	Participants (*N* = 32)
Age, median (range)	57 (30–79)
Female, No. (%)	26 (81)
Self-reported race/ethnicity, No. (%)	
African American/African	17 (53)
Hispanic/Latinx	15 (47)
Personal history of hATTR-related feature, No. (%)	18 (56)
Family history of hATTR-related feature, No. (%)	15 (47)

**Table 2:** Uptake of medical management recommendations among V142I variant positive individuals.

Follow-Up with Specialists (*N* = 32)	No. (%)	Weeks Post-Results Disclosure, Median (Range)
Cardiologist/Cardiovascular geneticist	18 (56)	4.9 (0.4–34.9)
Heart failure specialist	2 (6)	15.4 (1.9–28.9)
Neurologist	2 (6)	27.4 (16.0–38.7)
**Interventions among Individuals Seen by a Specialist (*N* = 16)**	No.* (%)	**Weeks Post-Results Disclosure, Median (Range)**
ECG	10 of 12 (83)	3.0 (0.4–20.0)
Echocardiogram	12 of 16 (75)	13.5 (2.1–41.0)
Tc-99m-PYP scintigraphy	10 of 13 (77)	8.3 (2.1–38.0)

* Five individuals had an electrocardiogram (ECG) and 2 had an echocardiogram in the 12 months preceding their specialist visit, and were not recommended additional ECGs or echocardiograms. Twelve individuals were recommended Tc-99m-PYP scintigraphy.

## Data Availability

All data from this study are presented in the main text or supplemental materials.

## Supplementary Materials

The following are available online at https://www.mdpi.com/2075-4426/11/1/49/s1, Table S1: Presence of hATTR-related systemic features and related symptoms of in 32 V142I variant positive individuals, Table S2: Presence of hATTR-related systemic features by age, sex, and self-reported race/ethnicity groups after follow-up with recommended specialists.
