# Supplementary material for: Genomic Screening Identifies Individuals at High Risk for Hereditary Transthyretin Amyloidosis
Source: J Pers Med. 2021 Jan 15;11(1):49. doi: 10.3390/jpm11010049 (PMC7829706; doi:10.3390/jpm11010049)
Supplement: Supplementary file 1 [file jpm-11-00049-s001.pdf]

Journal of Personalized Medicine

Supplementary Materials

Genomic screening identifies individuals at high risk for hereditary transthyretin amyloidosis

Emily R. Soper, MS[1,2#], Sabrina A. Suckiel, MS[1,2#], Giovanna T. Braganza, BS[1], Amy R. Kontorovich, MD, PhD[2,3], Eimear E. Kenny, PhD[1,2,4], Noura S. Abul-Husn, MD, PhD[1,2,4*]

[1] The Institute for Genomic Health, Icahn School of Medicine at Mount Sinai, New York, NY, USA
[2] Department of Medicine, Icahn School of Medicine at Mount Sinai, New York, NY, USA
[3] The Zena and Michael A. Wiener Cardiovascular Institute, Icahn School of Medicine, New York, NY, USA
[4] Department of Genetics and Genomic Sciences, Icahn School of Medicine at Mount Sinai, New York, NY, USA
[#] These authors contributed equally to this work.

* Correspondence: noura.abul-husn@mssm.edu

Table S1. Presence of hATTR-related systemic features and related symptoms in 32 V142I variant positive individuals.

Table S2. Presence of hATTR-related systemic features by age, sex, and self-reported ancestry after follow up with recommended specialists.

Citation: Soper, E.R.; Suckiel, S.A.; Braganza, G.T.; Kontorovich, A.R.; Kenny, E.E.; and Abul-Husn, N.S. Genomic screening identifies individuals at high risk for hereditary transthyretin amyloidosis. *J. Pers. Med.* **2021**, *11*, 49. https://doi.org/10.3390/jpm11010049

Received: 21 December 2020
Accepted: 09 January 2021
Published: 15 January 2021

Publisher's Note: MDPI stays neutral with regard to jurisdictional claims in published maps and institutional affiliations.

Copyright: © 2021 by the authors. Submitted for possible open access publication under the terms and conditions of the Creative Commons Attribution (CC BY) license (http://creativecommons.org/licenses/by/4.0/).

Table S1. Presence of hATTR-related systemic features and related symptoms in 32 V142I variant positive individuals.

**Phenotype**	**Systemic feature /** ***Related symptom***	**No. (%) at result disclosure**	**No. (%) after specialist follow-up**	**No. (%) with family history[*]**
Cardiac	Heart failure	4 (13)	5 (16)	6 (19)
	Cardiomyopathy	1 (3)	1 (3)	1 (3)
	Atrial fibrillation	0 (0)	1 (3)	3 (9) [†]
	Shortness of breath	16 (50)	19 (59)	-
	Dizziness	8 (25)	12 (38)	-
	Syncope	4 (13)	6 (19)	-
	Palpitations	9 (28)	12 (38)	-
	Edema	9 (28)	10 (31)	-
Peripheral neuropathy	Carpal tunnel syndrome	10 (31)	10 (31)	8 (25)
	Spinal stenosis	10 (31)	10 (31)	0 (0)
	Carpal tunnel-related symptoms	13 (41)	15 (47)	-
	Gait/Balance problems	11 (34)	13 (41)	-
	Muscle weakness	13 (41)	13 (41)	-
	Numbness/tingling	17 (53)	20 (63)	-
	Pain in extremities	14 (44)	14 (44)	-
	Back pain	16 (50)	18 (56)	-
	Temperature sensitivity	0 (0)	4 (13)	-
	Joint pain	18 (56)	19 (59)	-
Autonomic neuropathy	Autonomic dysfunction[#]	1 (3)	1 (3)	0 (0)
	Incontinence	4 (13)	5 (16)	0 (0)
	Sexual dysfunction/Impotence	3 (9)	4 (13)	0 (0)
	Nausea/vomiting	6 (19)	7 (22)	-
	Diarrhea	8 (25)	8 (25)	-
	Constipation	12 (38)	15 (47)	-
	Loss of appetite/early satiety	9 (28)	10 (31)	-
Presence of any systemic feature or related symptom		30 (94)	31 (97)	-
Presence of any systemic feature only		18 (56)	19 (59)	15 (47)

[*] Family history was assessed for presence of hATTR-related systemic features only.
[†] Family history was assessed broadly for arrhythmia and not specifically for atrial fibrillation.
[#] Autonomic dysfunction includes gastroparesis and orthostatic hypotension.

Table S2. Presence of hATTR-related systemic features by age, sex, and self-reported ancestry after follow up with recommended specialists.

Demographic variable	Presence of any hATTR-related systemic feature No. (%)	*P*-value
<60 years (N=18)	9 (50)	0.29
≥60 years (N=14)	10 (71)	
Female (N=26)	16 (62)	0.67
Male (N=6)	3 (50)	
African American/African (N=17)	9 (53)	0.49
Hispanic/Latinx (N=15)	8 (68)	
